# Plant invasion impacts on fungal community structure and function depend on soil warming and nitrogen enrichment

**DOI:** 10.1007/s00442-020-04797-4

**Published:** 2020-11-03

**Authors:** M. A. Anthony, K. A. Stinson, J. A. M. Moore, S. D. Frey

**Affiliations:** 1 Department of Natural Resources and the Environment, University of New Hampshire, Durham, NH 03824 USA; 2 Department of Environmental Conservation, University of Massachusetts, Amherst, MA 01001 USA; 3 Present Address: Bioscience Division, Oak Ridge National Laboratory, Oak Ridge, TN 37830 USA; 4 Present Address: Department of Environmental Systems Science, ETH Zürich, 8006 Zurich, Switzerland

**Keywords:** Arbuscular mycorrhizal fungi, Ectomycorrhizal fungi, Invasive species, Soil warming, Nitrogen deposition

## Abstract

The impacts of invasive species on biodiversity may be mitigated or exacerbated by abiotic environmental changes. Invasive plants can restructure soil fungal communities with important implications for native biodiversity and nutrient cycling, yet fungal responses to invasion may depend on numerous anthropogenic stressors. In this study, we experimentally invaded a long-term soil warming and simulated nitrogen deposition experiment with the widespread invasive plant *Alliaria petiolata* (garlic mustard) and tested the responses of soil fungal communities to invasion, abiotic factors, and their interaction. We focused on the phytotoxic garlic mustard because it suppresses native mycorrhizae across forests of North America. We found that invasion in combination with warming, but not under ambient conditions or elevated nitrogen, significantly reduced soil fungal biomass and ectomycorrhizal relative abundances and increased relative abundances of general soil saprotrophs and fungal genes encoding for hydrolytic enzymes. These results suggest that warming potentially exacerbates fungal responses to plant invasion. Soils collected from uninvaded and invaded plots across eight forests spanning a 4 °C temperature gradient further demonstrated that the magnitude of fungal responses to invasion was positively correlated with mean annual temperature. Our study is one of the first empirical tests to show that the impacts of invasion on fungal communities depends on additional anthropogenic pressures and were greater in concert with warming than under elevated nitrogen or ambient conditions.

**Electronic supplementary material:**

The online version of this article (10.1007/s00442-020-04797-4) contains supplementary material, which is available to authorized users.

## Introduction

Plant invasions are increasing at historically unprecedented rates (Seebens et al. 2018), and interactions with abiotic global changes may further promote establishment and spread of non-native species beyond their home ranges (Milchunas and Lauenroth 1995; Howard et al. 2004). The interactive effects of multiple anthropogenic global change drivers are widely recognized as important in determining future ecosystem functioning; however, less than 1% of all global change studies in soil ecology have tested interactions between biotic invasion (plant, animal, or microbes) and other global change factors (Rillig et al. 2019). Thus, we currently lack an empirical assessment of how belowground ecosystem structure and function respond to simultaneous invasion and abiotic global changes.

Most terrestrial plants host a belowground consortium of microorganisms, including fungi, which affect how ecosystems respond to invasion (Inderjit and van der Putten 2010). While most studies to date focus on plant responses to invasion, fungi are drivers of ecosystem nutrient cycling as decomposers and mycorrhizal symbionts (Treseder and Lennon 2015). How fungi respond to invasion can also feed-back to impact native plant communities (Stinson et al. 2006), soil carbon (C) storage (Ehrenfeld 2003; Tamura and Tharayil 2014), and ecosystem restoration efforts (Lankau et al. 2014; Anthony et al. 2019). Fungi are highly sensitive to abiotic stressors such as warming and nitrogen deposition (Lilleskov et al. 2011; Geml et al. 2015; Morrison et al. 2016; Fernandez et al. 2017), but the interactive effects of invasion and concurrent abiotic global changes on soil fungi are rarely investigated (Wheeler et al. 2017).

Here, we tested how two abiotic global changes of significance in the non-native range of *Alliaria petiolata* (garlic mustard), atmospheric nitrogen (N) deposition and soil warming, influence fungal community and functional responses to invasion. We focused on garlic mustard because it is invasive throughout temperate forests of North America (Rodgers et al. 2008), and on N deposition and warming because these anthropogenic pressures are co-occurring throughout the non-native range of garlic mustard (Galloway et al. 2004; Allen et al. 2018). Soil fungal biomass is reduced and community composition is altered by garlic mustard invasion due to it being non-mycorrhizal and producing antifungal phytochemicals (Rodgers et al. 2008; Barto et al. 2011; Lankau 2011). Garlic mustard particularly suppresses mycorrhizal fungi (Roberts and Anderson 2001; Stinson et al. 2006; Wolfe et al. 2008; Wheeler et al. 2017) which has been linked to reduced native plant diversity (Stinson et al. 2007) and growth (Stinson et al. 2006; Wheeler et al. 2017). The decline in mycorrhizal fungi with garlic mustard invasion is also associated with a shift towards increased saprotrophic and plant pathogenic fungal dominance (Anthony et al. 2017), but we do not understand how garlic mustard affects fungal communities and ecosystem function in the context of progressing global changes.

Our objective was to test whether the effects of garlic mustard invasion on soil fungi are ameliorated, unaffected, or amplified by conditions of soil N enrichment and warming. Typically, potential effects of invasion are inferred from changes in invasive species growth and cover across environmental conditions (Bradley et al. 2010; Merow et al. 2017). Previous work has shown that garlic mustard grows larger with N additions (Meekins and McCarthy 2000) but not under warmer temperatures (Anderson and Cipollini 2013), which suggests that N additions but not warming may amplify the impacts of invasion. However, the magnitude of the invasion effect will also depend on how N enrichment and warming influence fungal community resistance to invasion (i.e., capacity to be unchanged) which may be uncoupled from garlic mustard growth. To address this, we experimentally invaded a long-term soil warming and simulated N deposition experiment with garlic mustard and measured fungal community structure and function, including fungal growth, stress response, and cellulolytic and oxidative enzyme decomposition genes.

## Materials and methods

### Site description and experimental design

This work was conducted at the Soil Warming × Nitrogen Addition Study located at the Harvard Forest LTER in Petersham, MA (42°29′1′′ N 72°11′15′′ W). This experiment, initiated in 2006 to examine interactions between soil warming and simulated N deposition (see Contosta et al. 2011), is located in an even-aged, mixed deciduous stand with a canopy of red and black oak (*Quercus rubra*, *Q. velutina*), red and striped maple (*Acer rubrum*, *A. pensylvanicum*), American beech (*Fagus grandifolia*), white birch (*Betula papyrifera*), and an understory of saplings of the same species, along with stump sprouts of American chestnut (*Castanea denata*). Mean annual temperature at Harvard Forest is 8.3 °C and annual precipitation is 1247 mm (Boose and VanScoy 2001). Warming (5 °C above ambient) is achieved with heating cables buried to 10 cm, and simulated N deposition plots are fertilized with aqueous NH_4_–NO_3_ (5 g N m^−2^ year^−1^) at equal monthly doses throughout May–October.

In each of the plots (9 m^2^), we established uninvaded and invaded subplots (1 m^2^) using a fully factorial, randomly distributed design (*n* = 5; see diagram in Supplementary Fig. 1). First year garlic mustard seedlings were planted in invaded subplots in April 2015 at densities typical to the region (20 plants m^−2^) and allowed to establish and grow for approximately 1 year, then removed prior to the onset of reproduction. Though the size of the subplots is small, garlic mustard establishes in small, dense patches in temperate forest understories (Nuzzo 1999). To simulate a realistic invasion, the number of garlic mustard plants in each plot was maintained throughout the invasion at a similar density across treatments (see Wheeler et al. 2017 for details). To summarize, the full experiment had 4 main treatments (control, warming, N addition, N addition × warming), 2 invasion statuses (uninvaded, invaded), and 5 replicates for a total of 40 experimental units.

### Soil sampling and processing

Soil samples were collected a little over 1 year after the artificial invasion (July 2016) at two randomly selected locations within each plot. At each location, we removed a 100 cm^2^ block of the organic horizon to the depth of the mineral soil followed by a cylindrical core (5 cm width × 10 cm depth) of mineral soil. The two samples from each plot were homogenized by depth increment. Homogenized samples were stored on frozen ice packs in the field and immediately placed at 4 °C within 12 h of sampling. Samples were sieved (< 4 mm) within 24 h of sampling to remove roots, rocks, and organic debris. Subsamples for molecular (~ 2 g) and lipid (~ 10 g) analyses were taken from the sieved material and stored at − 80 °C and − 20 °C, respectively, until processing. The remaining soil was stored at 4 °C.

### Edaphic analyses

Soil pH was measured on soil slurries made from air-dried soil and deionized water (10 g soil: 20 mL deionized water). Total soil C and N were measured on dried, finely ground soils using an elemental analyzer (Perkin Elmer 2400 Series II CHN, Waltham, MA). Soil C stocks were calculated on a volumetric basis using bulk density measurements previously made at the site (unpublished data). Soil inorganic N concentrations were determined on 2 M extracts (10 g soil: 40 mL potassium chloride) using a colorimetric approach (Braman and Hendrix 1989), and net N mineralization was estimated by measuring inorganic N concentrations before and after a 7-day laboratory incubation and calculating the difference. In a separate incubation, we measured C mineralization using a 10-day laboratory incubation. Field moist soil (10 g) was incubated in Mason jars with lids equipped with sealed septa. The headspace of each jar was sampled daily and analyzed for CO_2_ concentration on a LICOR 6252 Infrared Gas Analyzer (LI-COR Biosciences, Lincoln, NE). After 10 days, we calculated an average respiration rate as a proxy for labile C. Fine root biomass was estimated by picking fine roots (≤ 2 mm) from 100 g of fresh mineral soil; fine root stocks were calculated on a volumetric basis using soil bulk density.

Fungal biomass was assessed via phospholipid and neutral lipid fatty acid analysis (P/N-LFA) for total fungi and AMF, respectively, consistent with Olsson et al. (1995). In short, lipids were extracted from freeze-dried soil (1 g) using phosphate buffer, chloroform, and methanol (0.8:1:2; v:v:v). The polar (phospholipids) and neutral lipids were isolated separately using silicic acid chromatography and methylated using 0.2 M methanolic potassium hydroxide (1 mL) at 60 °C for 30 min to form fatty acid methyl esters (FAMES) that were quantified on a Varian CP-3800 gas chromatograph equipped with a flame ionization detector (Agilent Technologies, Santa Clara, CA). We compared FAME peaks against a standard library of FAMES specific to fungi (18:2ω6, 9c, 18:1ω9c) and AMF (16:1ω5c). Standards for each marker were used to convert peak area concentrations to nmol PLFA/NLFA g^−1^ dry soil.

### Fungal community characterization

Fungal (including ectomycorrhizal fungi; EMF) and arbuscular mycorrhizal fungal (AMF) community structure was characterized using ITS2 and 18S metabarcoding on the Illumina MiSeq platform, respectively. DNA was extracted from soil (0.25 g) using the DNeasy PowerSoil Kit (Qiagen, Hilden, Germany). The ITS2 region was amplified using the fungal specific primer pair *f*ITS7 (Ihrmark et al. 2012) and ITS4 (White et al. 1990). Primers currently used to study fungi poorly cover the Glomeromycotina and therefore the 18S region was also amplified using the Glomeromycotina (AMF) specific primer pair NS31 (Simon et al. 1992) and AML2 (Lee et al. 2008). Going forward, we refer to fungi identified by the ITS2 region as ‘fungi’ and AMF identified by the 18S region as ‘AMF’. PCR primers contained the Illumina adaptor sequence, an 8 bp pad sequence, a 2 bp linker sequence, and were dual indexed to include two unique 8 bp sequences (see custom PCR primer constructs, Supplementary Table 1). PCR reactions were performed in triplicate for each sample in 25 µL reactions with the following reagents: PCR Grade H_2_O (13 µL), Phusion^®^ High-Fidelity PCR Master Mix with HF Buffer (10 µL; New England BioLabs Inc, Ipswitch, MA), 10 µM forward primer (0.5 µL), 10 µM reverse primer (0.5 µL), and template DNA (1 µL). Thermocycler conditions and library preparation followed that of Anthony et al. (2017). Equimolar libraries of the ITS2 and 18S samples were sequenced on two, replicate Illumina MiSeq v2 (2 × 250 bp) and two MiSeq v3 (2 × 300 bp) runs at the Center for Genomics and Bioinformatics at Indiana State University, Bloomington, IN, respectively. Different sequencing chemistries were used because the ITS2 amplicons are shorter than the 18S amplicons. Sequences are available in the NCBI database under the BioProject PRJNA522440 for ITS2 sequences and PRJNA522442 for 18S sequences.

All sequences were passed through a series of quality control measures. Illumina adapter and PCR primer sequences, reads < 100 bp, and low-quality bases and reads (Phred scores < 2) were removed using Trimmomatic (v0.32; Bolger et al. 2014). We then merged forward and reverse reads using the join_paired_ends.py function in QIIME (Caporaso et al. 2010). The ITS2 reads were merged at a 20 bp overlap allowing 5% mismatch. The 18S reads were merged at a 10 bp overlap allowing 10% mismatch. Merged ITS2 sequences were then passed through ITSx (Bengtsson‐Palme et al. 2013) to isolate the ITS2 region from flanking LSU and 5.8S regions. A total of 60% and 62% of initial paired end sequences were retained for the ITS2 and 18S datasets, respectively, after quality control (see Supplementary Table 2 for details on sequence retention). We used USEARCH (v8) to create OTU tables (Edgar 2010). Sequences were dereplicated (-fastx_uniques), sorted by size with singletons removed (-sortbysize), and clustered at 97% sequence similarity with chimera removal (-cluster_otus). We assigned taxonomy to ITS2 OTUs using the UNITE reference database (v7; Abarenkov et al. 2010) and the assign_taxonomy.py function in QIIME. 18S OTUs were blasted against the MARJAAM database (Öpik et al. 2010). OTUs without a match to fungi in the UNITE or MARJAAM databases were then blasted against the entire NCBI *nt* database, and OTUs assigned to non-fungal organisms for ITS2 data or non-Glomeromycotina fungi for the 18S sequences were removed from subsequent analyses to constrain analysis to the target groups. To confirm the taxonomy of the top 20 most common ITS2 fungi, we also performed manual BLAST searches and selected the best hit (lowest *E* value) if sequence identity was > 98%. Fungi from the ITS2 dataset were assigned guild annotations using FUNGuild and all “probable” or higher designations were included (Nguyen et al. 2016). Information on the number of OTUs and proportion of sequences assigned taxonomy and guild annotations is in Supplementary Table 2.

### Fungal functional characterization

To investigate fungal functional potential, we enriched and sequenced targeted functional genes. We focused on genes encoding hydrolytic extracellular enzymes (betaglucosidase, cellobiohydrolase, cellobioside dehydrogenase), oxidative extracellular enzymes (lignin peroxidase, manganese peroxidase, laccase, laccase-like multicopper oxidase), proteins used for general stress tolerance (RNA helicase, neutral trehalase; beta-1,3-glucan synthase; polyketide synthase), and ribosomal DNA production (partial 18S rRNA genes). The short, 18S rRNA gene sequences were used to investigate investments in processes such as growth since rRNA is frequently used as a marker for microbial biomass (Zhang et al. 2017) and rRNA levels increase during microbial growth (Klappenbach et al. 2000). We identified 2322 genes in NCBI related to the aforementioned fungal functions. Arbor Biosciences (Ann Arbor, MI) used these template sequences to design 34,249 candidate probes. Probe-to-probe complementary was diagnosed, and probes with > 94% similarity across > 83% of the sequences were clustered into a single representative of each cluster for the final probe set which consisted of 20,005 probes. The final custom probe set had a > 97% target gene enrichment success rate. Probes lengths were 100 nucleotides, and those < 100 nucleotides were padded with T’s. This length was chosen because of the relatively high GC content of the target sequences (average: 51%). Since we were targeting divergent regions of the fungal genome (i.e., length, similarity, rearrangements), probes were varied with 3 × tiling density. We used the myBaits Custom kit (Arbor Biosciences) and followed the manufacturer’s protocol to target and enrich genes.

DNA concentrations were diluted to 20 ng μL^−1^ and fragmented to 350 bp by sonicating (Covaris, Woburn, MA). Libraries were prepared for target probe enrichment using the Illumina TruSeq PCR-free Library Prep. Target-gene libraries were enriched in 50 μL reactions containing: template DNA (15 μL), 10 μM illumina flow site binding fragments (2.5 μL of P5 and P7), PCR master mix (25 μL; 2X Kapa HiFi HotStart ReadyMix, Kapa Biosystems), and PCR grade H_2_O (5 μL). Enriched libraries were then cleaned, quantified, and library prep was performed using the same methods as described above for the DNA metabarcoding libraries. The probe library was then sequenced on an Illumina Next-Seq 150 platform at the Center for Genomics and Bioinformatics at Indiana University (Bloomington, Indiana). Sequences are available in the NCBI database under the BioProject PRJNA633326.

Sequences were first passed through Trimmomatic to remove any Illumina adapter sequences and low-quality sequences (Phred < 2 at a 20 bp sliding window; < 1% sequences removed). Sequences were aligned using bwa (v7.17; Li et al. 2009) against a custom database constructed from the NCBI template DNA sequences. The probe database was created using the index algorithm, and forward and reverse reads were aligned using the bwa mem algorithm. We filtered alignments to include forward sequence alignments (retaining 59% of the sequences) because the forward and reverse reads did not pair. We only included alignments with > 90% sequence similarity using SAMtools (Li et al. 2009). A total of 38% of the sequences were aligned. Four samples were removed because of low sequencing depth. The remaining samples were rarified to the lowest sequence depth (9539 sequences per sample) using the rarefy function in vegan. We then calculated the relative abundance of individual genes and functional groups of genes encoding for hydrolytic enzymes, oxidative enzymes, stress tolerance, and growth.

### Statistical analyses

All statistical analyses were conducted in R (v3.6.1; R Core Team 2019) with criteria for rejecting the null hypothesis set to a *P *value of ≤ 0.05. Linear mixed effects models were used to assess whether the main effects of N addition, warming, and invasion, plus all interaction terms, had a significant effect on soil properties, soil processes, fungal biomass, and fungal functional guild relative abundances. We used the *lme* function adapted for type III sums of squares within the nlme package (Pinheiro et al. 2017). Since uninvaded and invaded subplots were within the same plot (i.e., split-plot design), plot was included in the model as a random effect. For all statistical models, we confirmed that residuals were normally distributed using Shapiro–Wilk tests of normality and we visually assessed qqnorm and residual versus fitted model plots.

To assess how much each treatment level effected the fungal community, we compared the effect size of each treatment level relative to uninvaded control plots. This allowed us to test whether the effect of invasion was enhanced, unchanged, or amplified by conditions of soil warming and N addition, which was our primary objective. We calculated effect sizes as Cohen’s *D* for each treatment level using the *cohen.d* function in the psych package (Revelle and Revelle 2015). Cohen’s *D* values of 0.2, 0.5, and 0.8 or greater represent small, medium, and large effect sizes, respectively. We also used Welch’s two-sample *t* tests with unequal variance and the base *t.test* function to assess whether independent treatment level differences were significant from uninvaded, control plots. Lastly, we calculated response ratios in treatment plots relative to uninvaded, control plots as log10 (Treatment_Value_/Control_Mean_) with 95% confidence intervals. Confidence intervals which did not pass through 0 were considered significant.

Variation in fungal communities across the abiotic global change treatments (nitrogen and warming) and invasion statuses were assessed on OTU relative abundances converted to Bray–Curtis dissimilarities using the *vegdist* function in vegan (Oksanen et al. 2013). The effects of nitrogen, warming, invasion, plus all two- and three-way interactions on fungal community composition were assessed using PERMANOVA implemented using the *adonis* function in vegan. To control for the effect of horizon, we included it as a random effect using the strata option. To compare treatment plots to uninvaded, control plots, we split the data-frame by treatment groups and performed pair-wise PERMANOVA comparisons. Lastly, we performed distance-based redundancy analysis (ds-RDA) using the *capscale* function (vegan) to visualize differences in fungal community composition using nitrogen, warming, and invasion as predictor variables.

## Results

### Fungal biomass and community composition

**Fig. 1:**
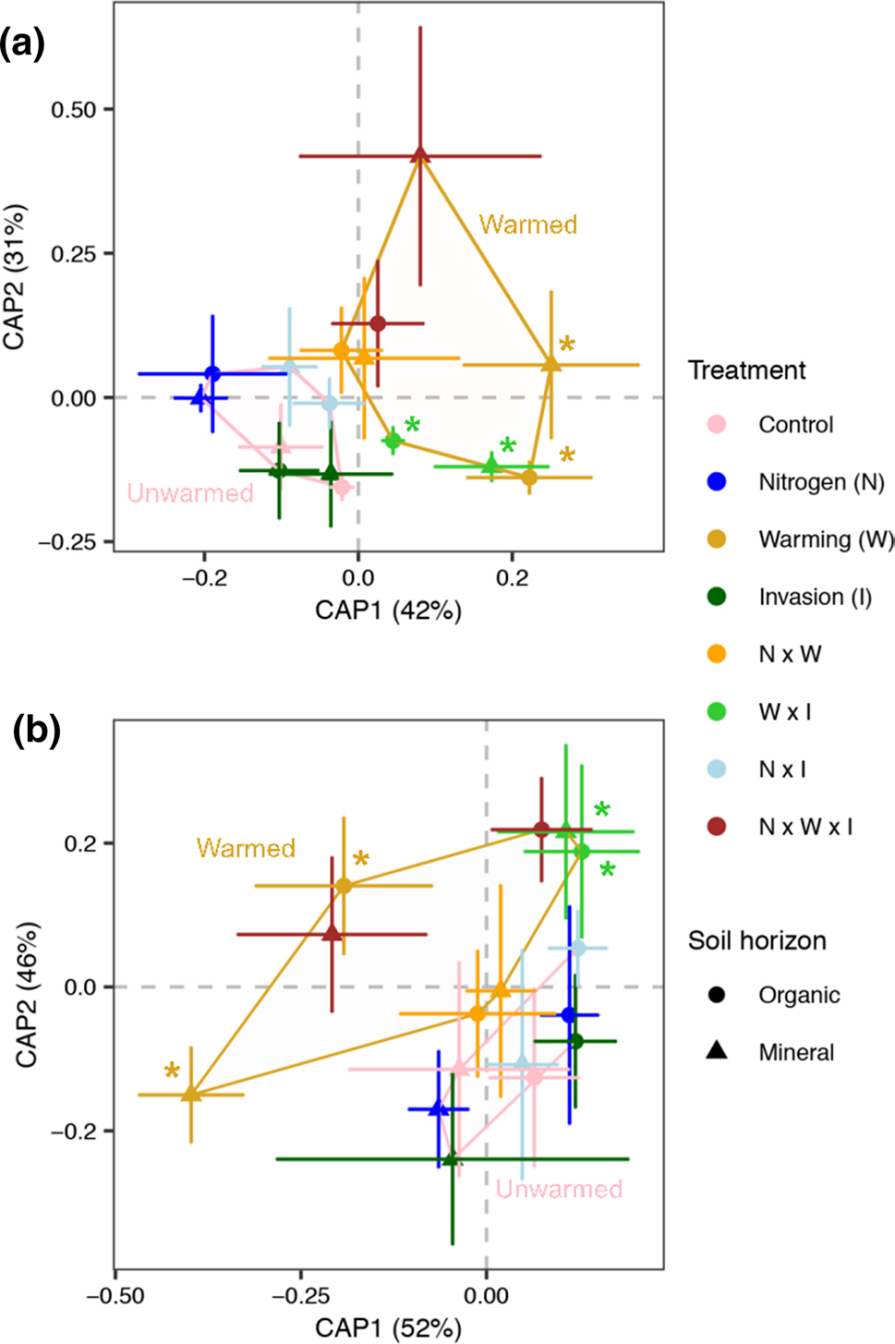
Fungal community composition across the global change treatments based on fungal ITS2 (**a**) and AMF 18S (**b**) DNA metabarcoding. Community composition was visualized using distance-based redundancy analysis (ds-RDA). Points represents the average site score configurations based on Bray–Curtis dissimilarity and error bars are the standard error. Pink and gold polygons outline the ranges of ambient temperature versus warmed plots which significantly differed from each other (*P*_ITS2_ = 0.008, *P*_18S_ = 0.004), respectively. Asterisks indicate significantly different treatment level community compositions relative to the uninvaded control plots (*P* ≤ 0.05; see Supplementary Table 4)

None of the global change factors affected fungal biomass individually, but there was a significant three-way interaction among N addition, warming, and invasion in the full statistical model in the organic horizon (*P* = 0.02; see mixed effects model results in Supplementary Table 3). Based on independent comparisons, fungal biomass was reduced by 43% in the two-factor warming × invasion plots in the organic horizon relative to control plots (Table 1). The effect size for fungal biomass was large in the warming × invasion plots (Cohen’s *D* = 2.1), particularly compared to the single factor invasion treatment (Cohen’s *D* = 0.44; Supplementary Fig. 2). Fungal community composition (ITS2) was unaffected by garlic mustard invasion and N additions alone, but it differed between ambient temperature and warmed plots (see polygons in Fig. 1a; *P* = 0.004), and there was also a significant three-way interaction among N addition, warming, and invasion in the full PERMANOVA model (*P* = 0.03; see PERMANOVA model results in Supplementary Table 4)*.* Treatment level comparisons relative to the uninvaded, control further revealed that fungal community composition was distinct in the single-factor warming and two-factor warming × invasion treatment (Supplementary Table 4).

**Table 1:** Soil properties and processes

Soil horizon	Treatment	Soil moisture (g H_2_O g^−1^ soil)	pH	Organic C (g C m^−2^)	C min (mg CO_2_ g^−1^ soil d^−1^)	Inorganic N (μg N g^−1^ soil)	N min (μg N g^−1^ soil d^−1^)	Fine root biomass (mg dry roots m^−2^)	Fungal biomass (nmol PLFA g^−1^ soil)	AMF biomass (nmol NLFA g^−1^ soil)
Organic	Control	0.51 (0.12)	4.0 (0.2)	2594 (252)	131 (31.4)	16.5 (6.6)	5 (1.2)	27 (7)	47 (6)	227 (26)
	Warming	0.46 (0.11)	4.2 (0.2)	**1640 (364)**	107 (62)	25.8 (7.4)	5.1 (2)	24 (1)	55 (15)	229 (45)
	Nitrogen	0.49 (0.11)	3.9 (0.1)	2107 (343)	128 (33)	28.1 (8.4)	5.4 (2.5)	23 (3)	59 (8)	285 (63)
	Invasion	0.54 (0.14)	4.0 (0.1)	2651 (730)	118 (43)	29.5 (8.8)	4.7 (1)	30 (5)	53 (9)	215 (47)
	Warming × nitrogen	0.40 (0.12)	4.2 (0.1)	2274 (609)	79 (39)	23 (2.8)	3.4 (1.4)	41 (17)	47 (8)	257 (93)
	Warming × invasion	0.37 (0.09)	4.3 (0.1)	1875 (447)	**48 (25)**	22.2 (4.6)	6.1 (2.2)	**48 (16)**	**27 (3)**	**79 (6)**
	Nitrogen × invasion	0.76 (0.09)	3.9 (0.1)	2706 (504**)**	153 (34)	24.8 (7.3)	6 (3.3)	**134 (5)**	43 (13)	295 (118)
	Warming × nitrogen × invasion	0.58 (0.08)	4.0 (0.2)	**1694 (408)**	89 (27)	16.8 (2.3)	3.1 (0.8)	34 (9)	56 (9)	210 (31)
Mineral	Control	0.26 (0.04)	4.3 (0.2)	3444 (200)	21 (8)	15.3 (2.9)	1 (0.5)	255 (59)	15 (2)	49 (27)
	Warming	0.26 (0.03)	4.3 (0.0)	3267 (133)	8 (4)	12.4 (3)	2.1 (0.8)	140 (17)	11 (3)	32 (13)
	Nitrogen	0.28 (0.03)	4.2 (0.1)	4327 (972)	11 (2)	18.1 (4)	1 (0.6)	661 (383)	13 (3)	64 (21)
	Invasion	0.32 (0.05)	4.2 (0.1)	3842 (273)	22 (6)	12.3 (2.1)	1.7 (0.6)	157 (30)	11 (3)	38 (11)
	Warming × nitrogen	0.28 (0.05)	4.3 (0.1)	3254 (515)	11 (3)	15.6 (2.4)	1.1 (0.5)	170 (28)	10 (2)	33 (10)
	Warming × invasion	0.24 (0.03)	4.4 (0.1)	3486 (545)	8 (3)	13.8 (4.8)	0.3 (0.2)	313 (184)	15 (4)	112 (54)
	Nitrogen × invasion	0.37 (0.01)	4.01 (0.1)	4124 (538)	13 (4)	10.4 (1.9)	0.7 (0.4)	87 (2)	26 (11)	108 (55)
	Warming × nitrogen × invasion	0.30 (0.03)	4.1 (0.2)	3782 (1512)	15.8 (3.1)	18.2 (1.6)	0 (0.3)	169 (6)	12 (1)	87 (25)

Values represent the mean, with standard errors in parentheses. Values in bold are significantly different from the control (*P* ≤ 0.05)

*C min* C mineralization measured using short-term laboratory incubations as a proxy for C lability, *N min* Net N mineralization measured using laboratory incubations

### Relative abundances of fungal functional and taxonomic groups

**Fig. 2:**
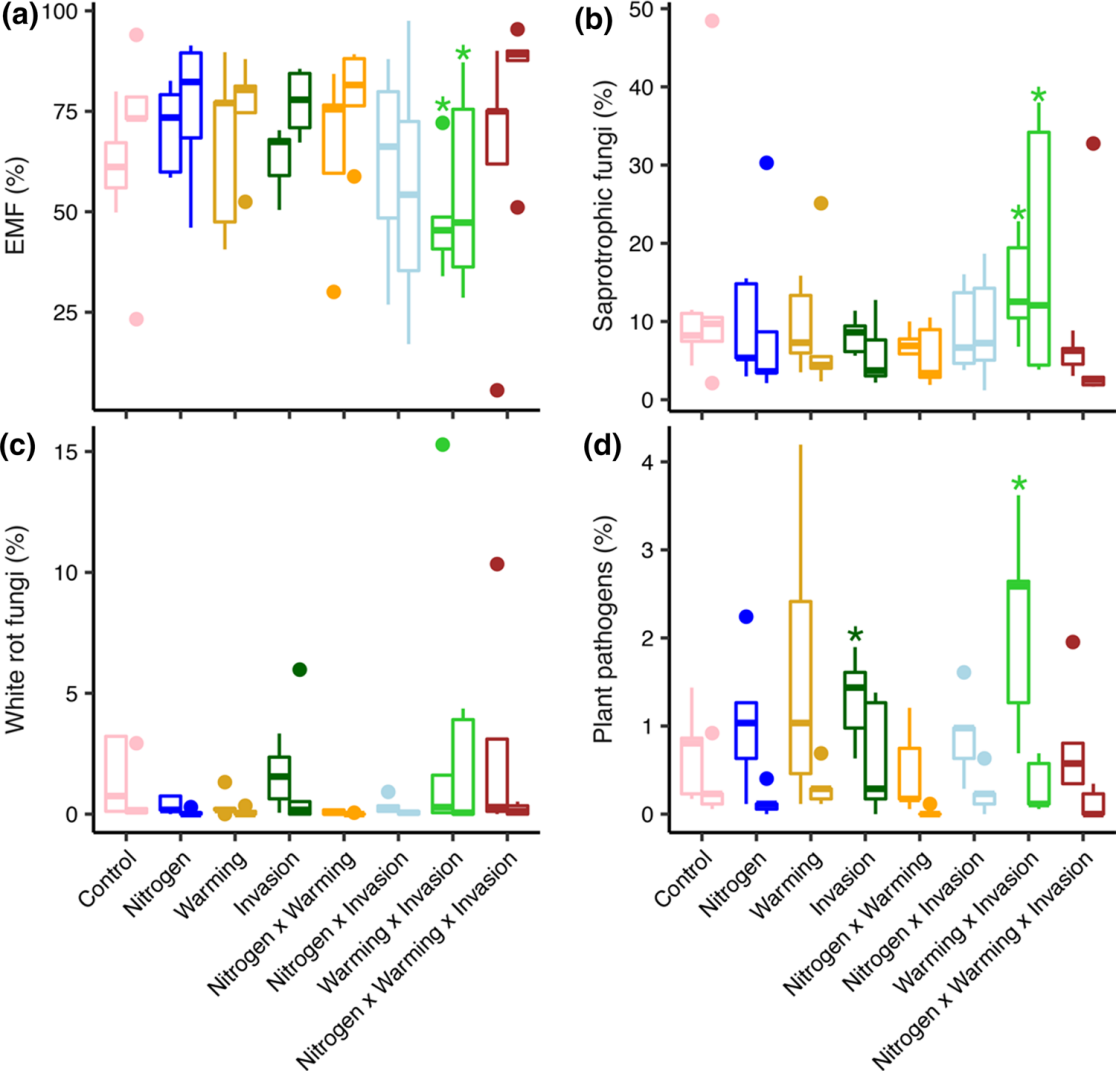
The relative abundance of fungal functional guilds. Relative abundances of ectomycorrhizal fungi (EMF), saprotrophic, white rot, and plant pathogenic fungi are plotted separately for organic (left bars) and mineral soil (right bars). Asterisks indicate significant differences relative to uninvaded, control plots (*P* ≤ 0.05). Note different *y*-axis limits on each panel

Ectomycorrhizal fungi were the dominant guild and comprised 48–83% of the total ITS2 sequences across treatment levels, though relative abundance was not impacted by N addition, invasion, or warming as single or interacting effects (Supplementary Table 5). However, independent comparisons relative to uninvaded, control plots revealed that the relative abundance of EMF was significantly reduced in the two-factor warming × invasion plots (Fig. 2a). The effect size for EMF relative abundance was large in the warming × invasion plots (Cohen’s *D* = 1.26), especially compared to the single-factor invasion treatment where the effect size was negligible (Cohen’s *D* = 0.02; Supplementary Fig. 3).

Saprotrophs were the second most dominant fungal guild, and while relative abundance was not affected by the single factor treatments, there was a significant three-way interaction effect on saprotroph relative abundance among N addition, warming, and invasion in the full statistical model in the organic horizon (*P* = 0.05) and between warming and invasion in the mineral soil (*P* = 0.02; see linear mixed effects model results in Supplementary Table 5). In comparison to the uninvaded control plots, the relative abundance of saprotrophs increased in relative abundance in the warming × invasion plots (Fig. 2b) and had a large effect size (Cohen’s *D* = 1.14). The third most dominant guild included plant pathogenic fungi which were not affected by the abiotic treatments as main effects, but increased in relative abundance in the two-factor warming × invasion treatment, though only significantly in the organic horizon (Fig. 2d). There were no shifts in the relative abundance of white rot fungi, the fourth most common guild.

**Fig. 3:**
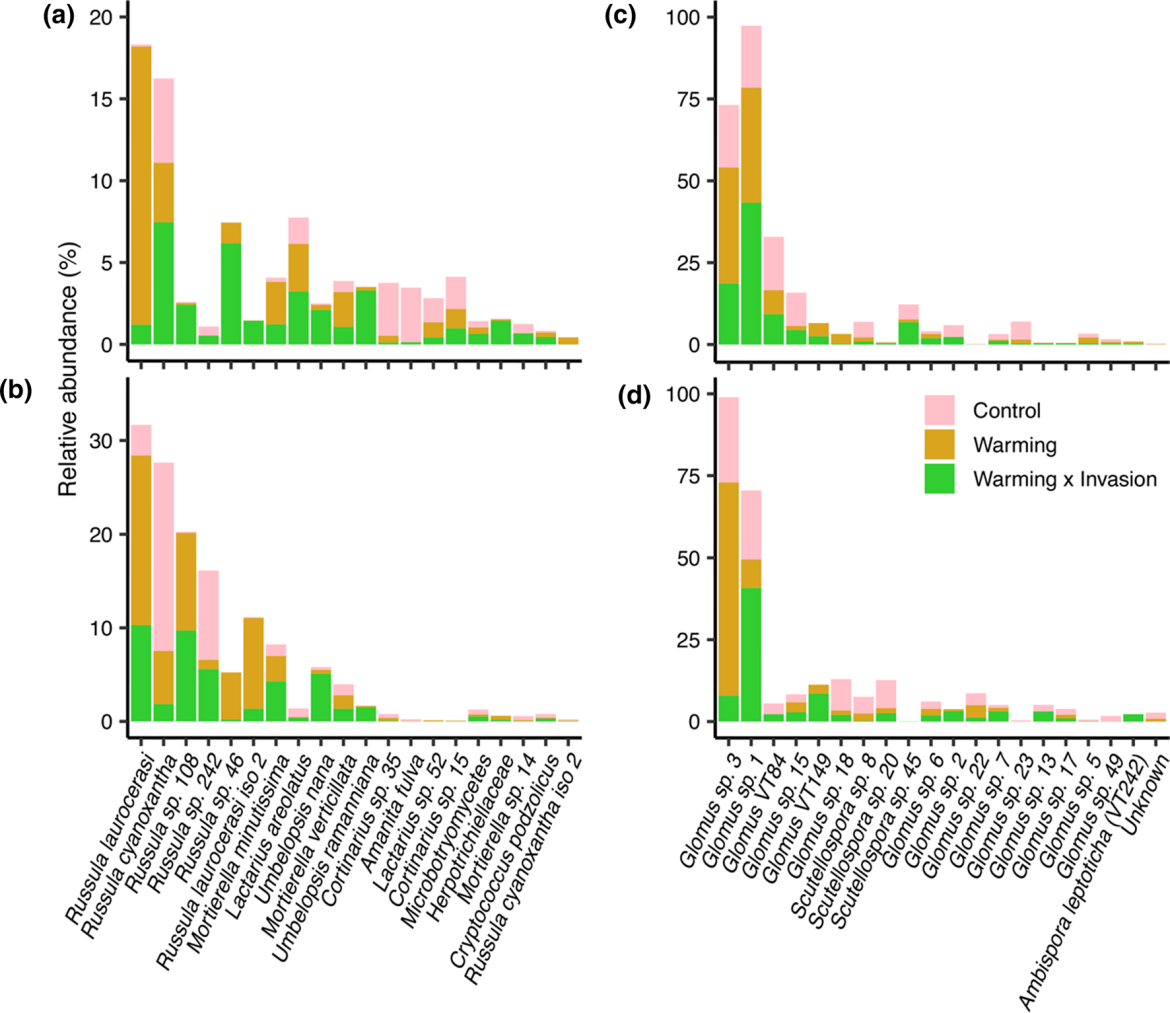
Rank abundance curves showing the top 20 most dominant fungal (**a**, **b**) and AMF (**c**, **d**) taxa. Bars represent the mean relative abundance. Only treatments where fungal community composition was significantly different from control are shown. Relative abundances are plotted separately for organic (**a**, **c**) and mineral (**b**, **d**) soil horizons

Relative abundances of the most dominant taxa, which were different EMF Russulaceae, varied in the single-factor and two-factor warming × invasion treatments relative to control plots (Fig. 3a, b). We only present the results for these two treatments for this fine-resolution analysis because fungal community composition exclusively differed from control plots for the warming and warming × invasion treatments. Different *Russula* lineages were dominant in control (*R. cyanoxantha*) versus warming only (*R. lauroceras*i) or warming × invasion plots (*Russula sp. 108*; see Supplementary Table 7), but *R. laurocerasi* was overly dominant in the single-factor warming plots (i.e., double the relative abundance in control plots; *P* = 0.04) and sensitive to the additional pressures of invasion. Three of the four most abundant saprotrophs (*Mortierella minutissima*, *Umbelopsis nana*, and* U. ramanniana*) were most abundant in the warming × invasion plots (Fig. 3a, b).

### AMF community biomass, composition, and taxonomic relative abundances

Arbuscular mycorrhizal fungal biomass was reduced by 65% in the organic horizon by the combined effects of warming and invasion (Table 1), but was not affected by invasion, N addition, or warming as single factor treatments (Supplementary Table 3). The effect size for AMF biomass was large (3.9), especially compared to the single-factor invasion treatment (0.15; Supplementary Fig. 2). AMF (18S) community composition differed between uninvaded and invaded plots as a single factor (*P* = 0.02) and between ambient temperature and warmed plots (see polygons in Fig. 1b; *P* = 0.001), but neither N addition nor the combined effects of N addition and warming had an effect (Supplementary Table 4). As observed for general fungi (ITS2), AMF communities were distinct from control in warming (*P* = 0.004) and warming × invasion plots (*P* = 0.02; Supplementary Table 4).

AMF within the *Glomus* genus were dominant and communities were hyper-uneven, with the two most dominant taxa having twofold higher relative abundances than the rest of the community (Fig. 3c, d). *Glomus* sp. 3 had twice the relative abundance in warming only compared to control plots (*P* = 0.01) and the warming × invasion plots (*P* = 0.002; Fig. 3; see Supplementary Table 7). Conversely, the relative abundance of *Glomus* sp. 1 was more than double the relative abundance in warming × invasion (43%; relative abundance) compared to control plots (21%; *P* = 0.02; Fig. 3c, d), and to a lesser extent warming alone (23%; *P* = 0.06). The second most common AMF genus, *Scutellospora*, did not vary with invasion or N addition, but it had reduced relative abundance with warming (3%) compared to control plots (12%; *P* = 0.03).

### Changes in fungal functional gene profiles

**Fig. 4:**
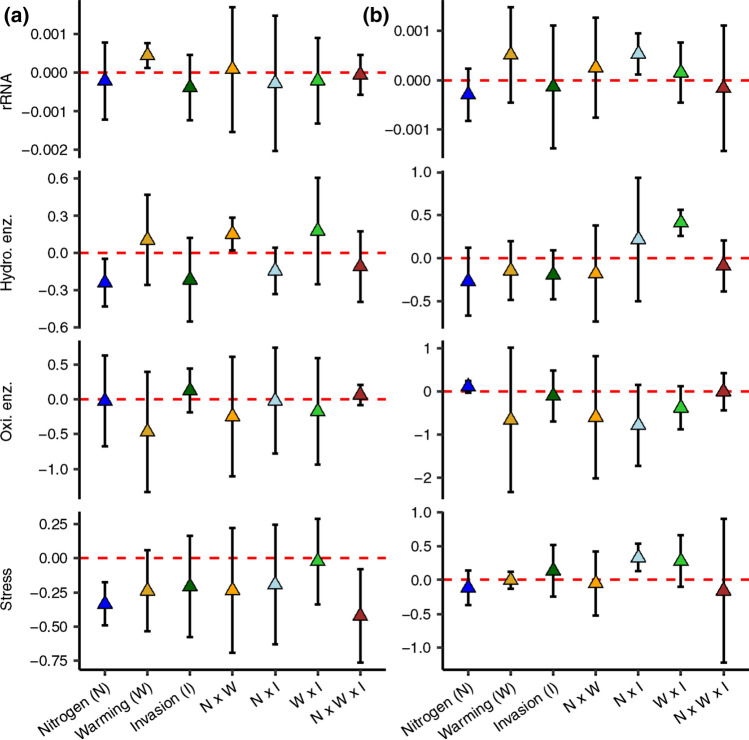
Response ratios of mean (± 95% CI) relative abundance of functional gene groups in treatment plots compared to control plots. Values which do not pass through 0 are significant at the 5% confidence level. *rRNA* ribosomal RNA, *Oxi. enz. *oxidative enzyme encoding genes, *Hydro. enz.* hydrolytic enzyme encoding genes

Fungal functional genes shifted in both the soil warming and N addition plots. The relative abundance of rRNA genes (a proxy for growth) was higher in the warming only treatment compared to uninvaded, control plots, but this was only significant in the organic horizon (*P* < 0.05; Fig. 4). Nitrogen addition alone reduced relative abundances of hydrolytic enzyme encoding and stress-response genes in the organic horizon. Nitrogen addition crossed with invasion increased rRNA and stress response gene proportions in mineral soil and increased hydrolytic enzyme encoding gene relative abundances in the organic horizon. Warming crossed with invasion also increased hydrolytic enzyme gene proportions, though only significantly in mineral soil.

### Soil properties and processes

Changes in soil properties and processes were generally small across the treatment plots with the exception of total soil C in the organic horizon. Neither invasion nor N addition as single-factors had an effect on soil properties or processes (Supplementary Table 3). Soil C was affected by warming in the organic horizon (*P* = 0.01), and specifically, was reduced compared to uninvaded, control plots in the single-factor warming and N addition × warming × invasion plots (Table 1). Carbon mineralization, a proxy for C lability, was reduced in the warming × invasion plots relative to control plots in the organic horizon. Neither, total inorganic N concentration nor net N mineralization were affected by N addition, warming, or invasion alone. Fine root biomass was not affected by warming or N addition, but organic horizon samples in the warming × invasion plots had more fine roots compared to uninvaded, control plots (Table 1).

## Discussion

As decomposers, mycorrhizal symbionts, and pathogens, soil fungi strongly shape the functioning of forested ecosystems (Treseder and Lennon 2015). What we know about their sensitivities to global change primarily comes from single-factor studies (Lekberg et al. 2007; Lilleskov et al. 2011; Morrison et al. 2016; Fernandez et al. 2017; Gibbons et al. 2017), but global change factors typically do not occur in isolation from each other (Aber et al. 2001). The impact of multiple global change stressors on microbial communities are seldom tested—only 20% of studies have examined more than one factor, and only 1% have examined more than two factors (Rillig et al. 2019). Our study addressed this gap by experimentally testing how soil fungal communities and their functional potential responded to simultaneous soil warming, N addition, and invasion by the non-native, phytotoxic plant, garlic mustard. Our study demonstrates that soil warming was the dominant factor to impact fungal communities and their functional capacity, but warming interacted with invasion and N addition. There was a significant three-way interaction effect among N addition, warming, and invasion in the full statistical models for fungal biomass and community composition; however, the effect size relative to uninvaded control plots was large and significant in the two-factor warming × invasion treatment but not in the single-factor invasion, two-factor nitrogen × invasion, or three-factor nitrogen × warming × invasion plots. This suggests that the impacts of garlic mustard invasion on soil fungi may be enhanced under warmer conditions but only in the absence of N additions.

### Warming altered fungal community structure and increased invasibility to garlic mustard

Garlic mustard invasion profoundly restructures temperate forest understories (Stinson et al. 2007; Rodgers et al. 2008) and belowground fungal communities (Barto et al. 2011; Lankau 2011) in North America. In part, this is related to the production of secondary chemicals (glucosinolates) which suppress native mycorrhizal fungi in North American forests where garlic mustard is invading (Stinson et al. 2006; Cantor et al. 2011). Surprisingly, we found no effect of invasion on soil fungi in absence of abiotic stressors. The impact of garlic mustard invasion on soil fungal communities can take years to manifest (Lankau 2011), which could explain why we saw no effect during our short-term (i.e., ~ 1 year) experimental invasion. However, in combination with warming, invasion reduced fungal and AMF biomass and altered the community composition of both groups. This indicates that fungi responded to a greater degree to multiple global change factors than single factors, and the community shaped by the abiotic filter of warming was especially susceptible to garlic mustard invasion.

Among plants, community composition can affect resistance to invasion if common species are sensitive to the invader (Wilsey and Polley 2002; Losure et al. 2007; Hillebrand et al. 2008). A similar form of resistance to invasion (or lack thereof) may apply to fungi as observed dominant fungi in the warming plots were sensitive to invasion whereas dominant fungi in the control plots were less responsive. The EMF, *Russula laurocerasi*, and the AMF, *Glomus* sp. 3, were both overly dominant in the warming plots (i.e., more than double the relative abundance of control plots), and the relative abundances of both taxa were reduced by warming × invasion. While we know little about the actual sensitivities of these taxa to invasion, warming within the temperature range of our study can stimulate fungal growth (Rillig et al. 2002; Pietikäinen et al. 2005). Faster growth reduces microbial ‘bet-hedging’ or the allocation of energy to unrequired growth conditions which facilitate adaptation to environmental changes (Mori et al. 2017; Kim et al. 2020). We observed that warming increased the relative abundance of fungal rRNA genes, a proxy for growth. Thus, one hypothesis is that faster growth in the warming plots reduced fungal capacities to adapt to garlic mustard invasion, leading to enhanced community turnover.

Long-term garlic mustard invasion can reduce fungal biomass (Cantor et al. 2011), EMF abundances (Wolfe et al. 2008), and shift fungal trophic guild dominance towards saprotrophic and plant pathogenic fungi (Anthony et al. 2017). While there were no differences in fungal functional group relative abundances in the invasion-only or invasion plus N addition plots, invasion in concert with warming reduced fungal biomass and relative abundance of EMF and increased that of saprotrophs and plant pathogens (Fig. 2). This mirrors expected results from previous research on long-term established invasions in the study region (Anthony et al. 2017, 2019). One possibility is that under warmer conditions garlic mustard invasion more strongly (or quickly) suppresses mutualistic fungi and selects for saprotrophic and pathotrophic taxa. As a result, under future warmer conditions, the phytotoxic effect of garlic mustard on forest fungi may be amplified, and forest soils may be more susceptible to new garlic mustard invasions than has been previously shown (Lankau 2011).

**Fig. 5:**
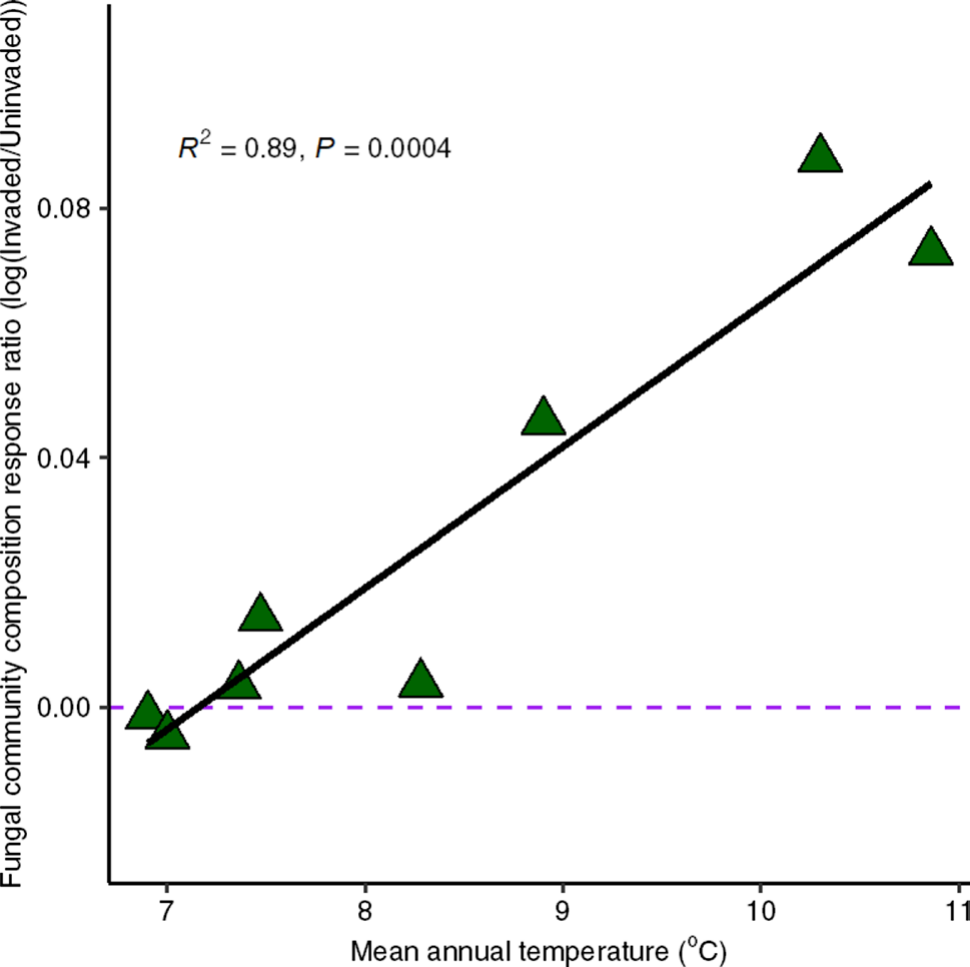
Dissimilarity in fungal community composition associated with garlic mustard invasion in relation to mean annual temperature (MAT). Responses were calculated as the log ratio of fungal community composition in plots actively invaded by garlic mustard to uninvaded plots across eight mixed temperate forests in the northeastern USA. Points represent mean configurations from non-metric multidimensional scaling [Data from Anthony et al. (2019)]

As a further test of the hypothesis that warming intensifies fungal responses to invasion, we examined whether fungal communities at sites with higher mean annual temperature (MAT) were more responsive to garlic mustard invasion compared to sites with lower MAT. We used previously published data on fungal communities from uninvaded and invaded plots across eight temperate forests in NY and MA (see Anthony et al. 2019). We calculated fungal community response ratios to garlic mustard invasion using non-metric multidimensional scaling configurations as log10 (invaded composition/uninvaded, control plot composition). We found a strong positive correlation between fungal community response to invasion and MAT over a temperature gradient of 4 °C (Fig. 5). This correlation could be due to other environmental variables, but when taken together with our experimental soil warming results, it provides another line of evidence supporting the hypothesis that warming intensifies fungal responses to invasion. It is also worth noting that the warming treatment in our experimental study was instrumented using buried heating cables. While this technique increases soil temperature, it does not appreciably warm aboveground (Aronson and McNulty 2009). Thus, the experimental effects we observed in the warming plots might be different, and possibly even stronger, if we warmed aboveground using infrared heating lamps.

### Nitrogen additions alone and in combination with invasion weakly affected fungal community composition

Nitrogen additions have well-known but variable effects on soil fungi (Frey et al. 2004; Lilleskov et al. 2011; Morrison et al. 2016; Linde et al. 2018). The impacts of N addition on fungi as a single-factor depend on the quantity of added N (Linde et al. 2018) and the duration of additions, and our study applied N at levels and over a time period (10 years) which do not always affect fungal community composition (Morrison et al. 2016). Nitrogen addition also had little effect on fungal community responses to invasion in the absence of warming. However, the effects of added N can potentially nullify the impacts of warming. For example, a recent meta-analysis found that warming elevates soil respiration and microbial biomass, but this effect is reversed by concurrent N additions (Yue et al. 2017). Here, we show that relative to control plots, communities were different in the single-factor warming treatment but when warming was crossed with N addition (N addition × warming; N addition × warming × invasion) no changes were observed, which supports the idea that N additions can mitigate the impacts of warming.

### Treatment effects on fungal functional genes, soil properties, and soil processes

In addition to fungal community shifts, there were differences in fungal functional profiles in response to warming and warming × invasion. The effects of warming on fungal functional potential varied by soil horizon: growth (rRNA) functional genes increased in relative abundance in the organic horizon where warming may be selecting for faster growing *r*-strategists (as discussed earlier), but this remains to be tested. In the two-factor warming × invasion plots, there was increased relative abundance of hydrolytic genes in mineral soil, which may promote the degradation of cellulose and beta-1,4, glucans (Ljungdahl and Eriksson 1985). Interestingly, warming × invasion promoted cellulose degrading genes and the saprotrophic fungi with fully sequenced genomes that were abundant in those plots possess > 10 copies of these cellulose degrading genes (Grigoriev et al. 2014). Our data suggest a higher potential for fungal growth in the warming plots and the enhanced degradation of cellulose in the warming × invasion treatment.

Nitrogen additions reduced the relative abundance of hydrolytic enzyme encoding genes but only as a single-factor treatment and in the organic horizon. Since soil organic matter decomposition in temperate forests is suppressed by N additions (Zak et al. 2008; Frey et al. 2014), this result is not unexpected. Relative abundance of stress response genes was also reduced by N additions, though only significantly in the organic horizon. Stress response genes were also at reduced relative abundance in the N addition × warming × invasion plots. There may be lower requirements for the enzymes encoded by these stress response genes under N additions because fungi are less metabolically active under chronic N limitations compared to ambient conditions (Frey et al. 2014), but this remains to be tested and would be limited to a certain N addition range as N eventually becomes toxic to microbes (Bowman et al. 2008). While we have demonstrated community composition and functional profile shifts specific to soil fungi, future studies should target particular fungal taxa that play disproportionate roles in mediating ecosystem responses to global changes. It is also important to acknowledge that whether these genes are being expressed is unknown. Future work on transcriptomes and proteomes remains to be done.

Physical soil characteristics were less sensitive to the treatments than were soil fungal communities, though there were changes in soil C stocks, C mineralization, fine root biomass and fungal biomass. Warming alone reduced total soil C stock, an expected outcome of elevated soil respiration under warming conditions (Contosta et al. 2011; Melillo et al. 2017). The observed higher relative abundance of rRNA genes under warming (Fig. 4) without a concomitant increase in fungal biomass (Table 1), further suggests shifts in fungal physiology including reduced growth efficiency. Warming can increase carbon allocation towards CO_2_ and away from biomass production (lower growth efficiency) and this can reduce soil C stocks (Frey et al. 2013). In the warming × invasion plots, carbon mineralization, a proxy for C lability, was reduced; fine root biomass increased; and total fungal and AMF biomass decreased in the organic horizon of the warming × invasion plots, suggesting that garlic mustard reduced a labile carbon source for fungi and fungal biomass via increased root in-growth. Fine root biomass was also higher in the organic horizons of the nitrogen × invasion plots, though this was not associated with other changes in soil properties/processes or fungal biomass. Garlic mustard has well known negative impacts on AMF (Cantor et al. 2011) and EMF biomass (Wolfe et al. 2008), and the effect of garlic mustard invasion on fungal biomass appeared to be amplified in the context of warming.

## Conclusion

This study shows that warming impacts how soil fungi respond to plant invasion but only in the absence of N additions. We acknowledge that it is possible that invasion modified the impact of warming as a single factor, but the observed fungal community shifts in mycorrhizal, saprotroph, and pathogen guilds were similar to those seen under long-term, established garlic mustard invasions. Reduced fungal and AMF biomass (Cantor et al. 2011), lower EMF relative abundance (Wolfe et al. 2008), and increased relative abundances of saprotrophs and pathogens (Anthony et al. 2017) are all associated with long-term garlic mustard invasions. Our observation of these effects within just 1 year of invasion in the warmed treatments but not under ambient conditions further suggests that warming may accelerate impacts of garlic mustard on forest fungi. Previously collected field data further support the idea that warming potentially exacerbates the invasion effect—across eight forested sites spanning a 4 °C temperature gradient, fungal community responses to invasion were positively correlated to MAT (Fig. 5). Garlic mustard invasion has substantial effects on temperate forest understories, and these impacts may worsen with projected increases in MAT in the northeastern USA.

## Electronic supplementary material

Below is the link to the electronic supplementary material.

Supplementary file1 (DOCX 20 KB)
