# Correction to: Plant invasion impacts on fungal community structure and function depend on soil warming and nitrogen enrichment

**DOI:** 10.1007/s00442-021-05079-3

**Published:** 2021-12-02

**Authors:** M. A. Anthony, K. A. Stinson, J. A. M. Moore, S. D. Frey

**Affiliations:** 1 Department of Natural Resources and the Environment, University of New Hampshire, Durham, NH 03824 USA; 2 Department of Environmental Conservation, University of Massachusetts, Amherst, MA 01001 USA; 3 Present Address: Bioscience Division, Oak Ridge National Laboratory, Oak Ridge, TN 37830 USA; 4 Present Address: Department of Environmental Systems Science, ETH Zürich, 8006 Zurich, Switzerland

## Correction to: Oecologia 10.1007/s00442-020-04797-4

Authors would like to correct the errors in Supplementary Table 1. The revised version of the Supplementary Table 1 is updated here.

The original article has been corrected.

## Supplementary Information

Below is the link to the electronic supplementary material.

Supplementary file1 (DOCX 19 kb)

