# Supplementary material for: Plant invasion impacts on fungal community structure and function depend on soil warming and nitrogen enrichment
Source: Oecologia. 2020 Nov 3;194(4):659–72. doi: 10.1007/s00442-020-04797-4 (PMC7683454; doi:10.1007/s00442-020-04797-4)
Supplement: Supplementary file 1 — Supplementary file1 (DOCX 20 KB) [file 442_2020_4797_MOESM1_ESM.docx]

**Supplementary Table 1.** Primer constructs designed for sequencing on Illumina MiSeq platforms and multiplexing using dual-barcoded *f*ITS7/NS31 and ITS4/AML2 primers

| **Name** | | **5' Illumina adapter / RC 3' Illumina adapter** | **Index** | | **Pad** | **Linker** | **PCR specific primer FITS7 or NS31**  **/ ITS4 or AML2** |
| --- | --- | --- | --- | --- | --- | --- | --- |
| SC501_FITS7 | | AATGATACGGCGACCACCGAGATCTACAC | ACGACGTG | | GCAGCGAGCC | GG | GTGARTCATCGAATCTTTG |
| SC502_FITS7 | | AATGATACGGCGACCACCGAGATCTACAC | ATATACAC | | GCAGCGAGCC | GG | GTGARTCATCGAATCTTTG |
| SC503_FITS7 | | AATGATACGGCGACCACCGAGATCTACAC | CGTCGCTA | | GCAGCGAGCC | GG | GTGARTCATCGAATCTTTG |
| SC504_FITS7 | | AATGATACGGCGACCACCGAGATCTACAC | CTAGAGCT | | GCAGCGAGCC | GG | GTGARTCATCGAATCTTTG |
| SC505_FITS7 | | AATGATACGGCGACCACCGAGATCTACAC | GCTCTAGT | | GCAGCGAGCC | GG | GTGARTCATCGAATCTTTG |
| SC506_FITS7 | | AATGATACGGCGACCACCGAGATCTACAC | GACACTGA | | GCAGCGAGCC | GG | GTGARTCATCGAATCTTTG |
| SC507_FITS7 | | AATGATACGGCGACCACCGAGATCTACAC | TGCGTACG | | GCAGCGAGCC | GG | GTGARTCATCGAATCTTTG |
| SC508_FITS7 | | AATGATACGGCGACCACCGAGATCTACAC | TAGTGTAG | | GCAGCGAGCC | GG | GTGARTCATCGAATCTTTG |
| SD501_FITS7 | | AATGATACGGCGACCACCGAGATCTACAC | AAGCAGCA | | GCAGCGAGCC | GG | GTGARTCATCGAATCTTTG |
| SD502_FITS7 | | AATGATACGGCGACCACCGAGATCTACAC | ACGCGTGA | | GCAGCGAGCC | GG | GTGARTCATCGAATCTTTG |
| SD503_FITS7 | | AATGATACGGCGACCACCGAGATCTACAC | CGATCTAC | | GCAGCGAGCC | GG | GTGARTCATCGAATCTTTG |
| SD504_FITS7 | | AATGATACGGCGACCACCGAGATCTACAC | TGCGTCAC | | GCAGCGAGCC | GG | GTGARTCATCGAATCTTTG |
| SD505_FITS7 | | AATGATACGGCGACCACCGAGATCTACAC | GTCTAGTG | | GCAGCGAGCC | GG | GTGARTCATCGAATCTTTG |
| SD506_FITS7 | | AATGATACGGCGACCACCGAGATCTACAC | CTAGTATG | | GCAGCGAGCC | GG | GTGARTCATCGAATCTTTG |
| SD507_FITS7 | | AATGATACGGCGACCACCGAGATCTACAC | GATAGCGT | | GCAGCGAGCC | GG | GTGARTCATCGAATCTTTG |
| SD508_FITS7 | | AATGATACGGCGACCACCGAGATCTACAC | TCTACACT | | GCAGCGAGCC | GG | GTGARTCATCGAATCTTTG |
| SD701_ITS4 | | CAAGCAGAAGACGGCATACGAGAT | ACCTAGTA | | GGTCTGCGCG | AA | TCCTCCGCTTATTGATATGC |
| SD702_ITS4 | | CAAGCAGAAGACGGCATACGAGAT | ACGTACGT | | GGTCTGCGCG | AA | TCCTCCGCTTATTGATATGC |
| SD703_ITS4 | | CAAGCAGAAGACGGCATACGAGAT | ATATCGCG | | GGTCTGCGCG | AA | TCCTCCGCTTATTGATATGC |
| SD704_ITS4 | | CAAGCAGAAGACGGCATACGAGAT | CACGATAG | | GGTCTGCGCG | AA | TCCTCCGCTTATTGATATGC |
| SD705_ITS4 | | CAAGCAGAAGACGGCATACGAGAT | CGTATCGC | | GGTCTGCGCG | AA | TCCTCCGCTTATTGATATGC |
| SD706_ITS4 | | CAAGCAGAAGACGGCATACGAGAT | CTGCGACT | | GGTCTGCGCG | AA | TCCTCCGCTTATTGATATGC |
| SD707_ITS4 | | CAAGCAGAAGACGGCATACGAGAT | GCTGTAAC | | GGTCTGCGCG | AA | TCCTCCGCTTATTGATATGC |
| SD708_ITS4 | | CAAGCAGAAGACGGCATACGAGAT | GGACGTTA | | GGTCTGCGCG | AA | TCCTCCGCTTATTGATATGC |
| SD710_ITS4 | | CAAGCAGAAGACGGCATACGAGAT | TAAGTCTC | | GGTCTGCGCG | AA | TCCTCCGCTTATTGATATGC |
| SD711_ITS4 | | CAAGCAGAAGACGGCATACGAGAT | TACACAGT | | GGTCTGCGCG | AA | TCCTCCGCTTATTGATATGC |
| SD712_ITS4 | | CAAGCAGAAGACGGCATACGAGAT | TTGACGCA | | GGTCTGCGCG | AA | TCCTCCGCTTATTGATATGC |
| NS31_SA1 | | AATGATACGGCGACCACCGAGATCTACAC | ATCGTACG | | TATGGTAATT | GC | TTGGAGGGCAAGTCTGGTGCC |
| NS31_SA2 | | AATGATACGGCGACCACCGAGATCTACAC | ACTATCTG | | TATGGTAATT | GC | TTGGAGGGCAAGTCTGGTGCC |
| NS31_SA3 | | AATGATACGGCGACCACCGAGATCTACAC | TAGCGAGT | | TATGGTAATT | GC | TTGGAGGGCAAGTCTGGTGCC |
| NS31_SA4 | | AATGATACGGCGACCACCGAGATCTACAC | CTGCGTGT | | TATGGTAATT | GC | TTGGAGGGCAAGTCTGGTGCC |
| NS31_SB1 | | AATGATACGGCGACCACCGAGATCTACAC | CTACTATA | | TATGGTAATT | GC | TTGGAGGGCAAGTCTGGTGCC |
| NS31_SB2 | | AATGATACGGCGACCACCGAGATCTACAC | CGTTACTA | | TATGGTAATT | GC | TTGGAGGGCAAGTCTGGTGCC |
| NS31_SB3 | | AATGATACGGCGACCACCGAGATCTACAC | AGAGTCAC | | TATGGTAATT | GC | TTGGAGGGCAAGTCTGGTGCC |
| NS31_SB4 | | AATGATACGGCGACCACCGAGATCTACAC | TACGAGAC | | TATGGTAATT | GC | TTGGAGGGCAAGTCTGGTGCC |
| NS31_SC1 | | AATGATACGGCGACCACCGAGATCTACAC | ACGACGTG | | TATGGTAATT | GC | TTGGAGGGCAAGTCTGGTGCC |
| NS31_SC2 | | AATGATACGGCGACCACCGAGATCTACAC | ATATACAC | | TATGGTAATT | GC | TTGGAGGGCAAGTCTGGTGCC |
| NS31_SC3 | | AATGATACGGCGACCACCGAGATCTACAC | CGTCGCTA | | TATGGTAATT | GC | TTGGAGGGCAAGTCTGGTGCC |
| NS31_SC4 | | AATGATACGGCGACCACCGAGATCTACAC | CTAGAGCT | | TATGGTAATT | GC | TTGGAGGGCAAGTCTGGTGCC |
| NS31_SD1 | | AATGATACGGCGACCACCGAGATCTACAC | AAGCAGCA | | TATGGTAATT | GC | TTGGAGGGCAAGTCTGGTGCC |
| NS31_SD2 | | AATGATACGGCGACCACCGAGATCTACAC | ACGCGTGA | | TATGGTAATT | GC | TTGGAGGGCAAGTCTGGTGCC |
| NS31_SD3 | | AATGATACGGCGACCACCGAGATCTACAC | CGATCTAC | | TATGGTAATT | GC | TTGGAGGGCAAGTCTGGTGCC |
| NS31_SD4 | | AATGATACGGCGACCACCGAGATCTACAC | TGCGTCAC | | TATGGTAATT | GC | TTGGAGGGCAAGTCTGGTGCC |
| AML2_SA1 | | CAAGCAGAAGACGGCATACGAGAT | AACTCTCG | | GGTCTGCGCG | GA | GAACCCAAACACTTTGGTTTCC |
| AML2_SA2 | | CAAGCAGAAGACGGCATACGAGAT | ACTATGTC | | GGTCTGCGC | GA | GAACCCAAACACTTTGGTTTCC |
| AML2_SA3 | | CAAGCAGAAGACGGCATACGAGAT | AGTAGCGT | | GGTCTGCGCG | GA | GAACCCAAACACTTTGGTTTCC |
| AML2_SA4 | | CAAGCAGAAGACGGCATACGAGAT | CAGTGAGT | | GGTCTGCGCG | GA | GAACCCAAACACTTTGGTTTCC |
| AML2_SB1 | | CAAGCAGAAGACGGCATACGAGAT | AAGTCGAG | | GGTCTGCGCG | GA | GAACCCAAACACTTTGGTTTCC |
| AML2_SB2 | | CAAGCAGAAGACGGCATACGAGAT | ATACTTCG | | GGTCTGCGCG | GA | GAACCCAAACACTTTGGTTTCC |
| AML2_SB3 | | CAAGCAGAAGACGGCATACGAGAT | AGCTGCTA | | GGTCTGCGCG | GA | GAACCCAAACACTTTGGTTTCC |
| AML2_SB4 | | CAAGCAGAAGACGGCATACGAGAT | CATAGAGA | | GGTCTGCGCG | GA | GAACCCAAACACTTTGGTTTCC |
| AML2_SC1 | | CAAGCAGAAGACGGCATACGAGAT | ACCTACTG | | GGTCTGCGCG | GA | GAACCCAAACACTTTGGTTTCC |
| AML2_SC2 | | CAAGCAGAAGACGGCATACGAGAT | AGCGCTAT | | GGTCTGCGCG | GA | GAACCCAAACACTTTGGTTTCC |
| AML2_SC3 | | CAAGCAGAAGACGGCATACGAGAT | AGTCTAGA | | GGTCTGCGCG | GA | GAACCCAAACACTTTGGTTTCC |
| AML2_SC4 | | CAAGCAGAAGACGGCATACGAGAT | CATGAGGA | | GGTCTGCGCG | GA | GAACCCAAACACTTTGGTTTCC |
| AML2_SD1 | | CAAGCAGAAGACGGCATACGAGAT | ACCTAGTA | | GGTCTGCGCG | GA | GAACCCAAACACTTTGGTTTCC |
| AML2_SD2 | | CAAGCAGAAGACGGCATACGAGAT | ACGTACGT | | GGTCTGCGCG | GA | GAACCCAAACACTTTGGTTTCC |
| AML2_SD3 | | CAAGCAGAAGACGGCATACGAGAT | ATATCGCG | | GGTCTGCGCG | GA | GAACCCAAACACTTTGGTTTCC |
| AML2_SD4 | | CAAGCAGAAGACGGCATACGAGAT | CACGATAG | | GTCTGCGC | GA | GAACCCAAACACTTTGGTTTCC |
| **Read 1 (NS31) sequence primer (Tm = 65.8)** | | |  |  |  |  |  |
| TATGGTAATT GC TTGGAGGGCAAGTCTGGTGCC | | |  |  |  |  |  |
| **Read 2 (AML2) sequence primer (Tm = 67.7)** | | |  |  |  |  |  |
| GGTCTGCGCG GA GAACCCAAACACTTTGGTTTCC | | |  |  |  |  |  |
| **Index (NS31-AML2) sequencing primer (Tm = 67.7)** | | |  |  |  |  |  |
| GGAAACCAAAGTGTTTGGGTTC TC CGCGCAGACC | | |  |  |  |  |  |
| **Read 1 (fITS7) sequencing primer (Tm = 66.1)** | | |  |  |  |  |  |
| GCAGCGAGCCGGGTGARTCATCGAATCTTTG | | |  |  |  |  |  |
| **Read 2 (ITS4) sequencing primer (Tm = 64.9)** | | |  |  |  |  |  |
| GGTCTGCGCGAATCCTCCGCTTATTGATATGC | | |  |  |  |  |  |
| **Index (fITS7-ITS4) sequencing primer (Tm = 66.3)** | | |  |  |  |  |  |
| GCATATCAATAAGCGGAGGATTCGCGCAGACC | | |  |  |  |  |  |
